# Exploratory Analysis of Electrical Impedance Tomography Features Associated with Spirometric Airflow Limitation

**DOI:** 10.34133/csbj.0214

**Published:** 2026-09-01

**Authors:** Anzhelika Mezina, Samuel Genzor, Stepan Miklanek, Jan Mizera, Tomas Krejci, Ladislav Stanke, Radim Burget, Aleksandr Ometov

**Affiliations:** ^1^ Dept. of Telecommunications, FEEC, Brno University of Technology, Brno 616 00, Czech Republic.; ^2^ Department of Respiratory Medicine, University Hospital Olomouc and Faculty of Medicine and Dentistry, Palacký University Olomouc, Olomouc 779 00, Czech Republic.; ^3^ Faculty of Medicine and Dentistry, Palacký University Olomouc, Olomouc 779 00, Czech Republic.; ^4^ The Czech National E-health Center, University Hospital Olomouc, Olomouc 779 00, Czech Republic.; ^5^ Tampere Wireless Research Center, Electrical Engineering Unit, Faculty of Information Technology and Communication Sciences, Tampere University, Tampere 33720, Finland.

## Abstract

**Introduction:** Electrical impedance tomography (EIT) is a noninvasive, radiation-free imaging modality that provides real-time information on regional lung ventilation from wearable sensors. Chronic obstructive pulmonary disease (COPD) is a highly prevalent respiratory disease causing persistent and progressive airway obstruction. The diagnosis is based primarily on spirometry, in which a Tiffeneau index (forced expiratory volume in 1 s/forced vital capacity ratio) below 0.7 is a key feature of the definitive diagnosis. Despite many advances in medicine, there is a lack of widely available methods for estimating airway obstruction using noninvasive bedside measurements, which could facilitate assessment in patients who are unable to perform standard spirometry, e.g., patients after laryngectomy and those with chronic tracheostomies. Moreover, the prevalence of COPD in this group of patients may be substantial. **Methods:** The study examined the relationship between EIT-derived signal features and the Tiffeneau index through a comprehensive statistical and machine learning analysis of patient data collected using the Dräger EIT system. Data from 15 adult patients who successfully completed conventional spirometry and EIT measurements were analyzed. Patients conducted the examination a couple of times; consequently, 30 measurements were collected. A total of 755 time-series features, complemented by physiological measurements, were extracted, analyzed, and evaluated using correlation metrics, *P*-value testing, categorical associations, and Shapley additive explanations explainability. **Results:** Frequency-domain features (fast Fourier transform angle coefficients), entropy measures, and autocorrelation-based descriptors have the strongest associations with the Tiffeneau index. Feature-selected machine learning models demonstrated that EIT-derived time–frequency features, combined with anthropometric variables (weight and body mass index), could approximate Tiffeneau index values in this small cohort (best model: $R^{2}=0.72$ on the testing set). **Conclusion:** These findings support the exploratory feasibility of EIT-based, noninvasive approaches for pulmonary function estimation in a general clinical cohort and motivate future validation studies in patients for whom spirometry cannot be performed.

## Introduction

Electrical impedance tomography (EIT) is a noninvasive, radiation-free technique used to monitor lung function in real time. It measures regional lung ventilation distribution by assessing impedance changes within the lungs [1]. EIT provides valuable information for optimizing mechanical ventilation and assessing pulmonary perfusion. This enables clinicians to assess regional lung function, detect ventilation imbalances, and inform treatment strategies, particularly in critically ill or mechanically ventilated patients [2]. The noninvasive nature is especially beneficial in intensive care settings, where patients may be sedated, unconscious, or mechanically ventilated. EIT enables continuous bedside monitoring of lung ventilation and perfusion, allowing clinicians to make informed decisions about ventilator settings and other therapeutic interventions [2]. Modern EIT is still executed in clinical settings in hospitals, but the development toward more mobile wearable systems is underway [3,4]. In addition, EIT in an awake patient may be a potentially diagnostic tool for lung function measurement. Chronic obstructive pulmonary disease (COPD) is a prevalent disease characterized by persistent airway limitation [5]. The obstruction is defined by a reduction in the Tiffeneau index (ratio of forced expiratory volume in 1 s [FEV1] to forced vital capacity [FVC]) below 0.7 (70%), measured by spirometry. Not all patients are able to undergo standard spirometry. For instance, many individuals with chronic tracheostomy following laryngectomy or those with severe malformations or mutilations of the splanchnocranium may find it challenging to measure pulmonary function. In such cases, achieving an accurate and comprehensive diagnosis can be nearly impossible. Additionally, a considerable risk factor associated with some conditions that lead to chronic tracheostomy, such as laryngeal cancer, is the prevalence of COPD, which can be as high as 30%, compared to approximately 8% in the general population [6]. To address this diagnostic gap, EIT is increasingly being explored as a noninvasive alternative for estimating spirometric parameters and assessing regional lung function [7]. Recent studies have demonstrated the clinical utility of EIT in detecting regional ventilation abnormalities and phenotyping diseases such as COPD and preserved ratio impaired spirometry [8], as well as its adaptability in various clinical and home settings via self-administrable, portable systems [9]. Optimization of EIT protocols, such as adjusting EIT belt positions, has also been shown to improve signal quality and preserve ventilation signals under challenging physiological conditions [10]. Building on these functional assessments, researchers have begun correlating EIT-derived signals directly with standard spirometry measures. Early analytical approaches focused on extracting frequency components, spectral features, and tidal volume changes to estimate FVC, FEV1, and the Tiffeneau index [11–13]. More recently, there has been a shift toward integrating these temporal and spectral features into machine learning (ML) and deep learning frameworks. Advanced models have been utilized to estimate respiratory parameters directly from raw EIT time-series data using recurrent networks [14], improve image and shape reconstruction algorithms [15,16], and automate the prediction of lung function indices using feature selection on frequency and entropy-based characteristics [17,18]. Furthermore, artificial intelligence (AI)-driven prediction models have been applied to COPD-related outcomes [19], incorporating anthropometric and clinical variables such as body mass index (BMI) as key predictors alongside physiological signals. Despite the growing number of studies demonstrating that EIT can capture ventilation-related information and correlate with conventional pulmonary function parameters, several critical gaps remain. Previous EIT-based spirometry studies frequently focus on predictive accuracy in healthy cohorts [13], rely on simultaneous EIT–spirometry calibration [11], or utilize black-box learning models that lack clinical interpretability [14]. In contrast, the clinical relevance of EIT becomes particularly strong in scenarios where spirometry cannot be reliably performed, such as in patients with altered airway anatomy after laryngectomy or with long-term tracheostomy. There remains a need for systematic, interpretable methods that identify exactly which aspects of EIT breathing signals, derived purely from time-series data without forced maneuvers, are most strongly associated with airflow obstruction. To address this gap, our work performed large-scale time-series feature extraction from global tidal EIT signals and combined statistical association analysis, ML-based prediction, and explainability techniques to both estimate obstruction severity via the Tiffeneau index and reveal the dominant underlying signal characteristics driving this relationship. Unlike previous EIT-based spirometry studies that focused purely on predictive accuracy, our work addresses patients unable to perform spirometry by analyzing EIT time-series data to identify key physiological signal patterns, particularly phase-related fast Fourier transform (FFT) coefficients, entropy measures, and autocorrelation descriptors. While this study focuses on cases where spirometry is impractical (e.g., postlaryngectomy patients), it currently draws from data of patients who completed standard spirometry. The main contributions of this paper are as follows:

• The study analyzed 755 EIT features using Pearson correlation, categorical correlation ratio, and *P*-value testing, revealing strong associations between lung function impairment and spectral, entropy-based, and autocorrelation features.
• The analysis highlights that phase-related FFT coefficients, energy-ratio metrics, and signal-complexity measures are particularly informative for estimating FEV1/FVC from EIT, even without spirometry data.
• Multiple ML algorithms were evaluated using cross-validated hyperparameter optimization, demonstrating that nonlinear models, i.e., multilayer perceptron (MLP), are well suited for capturing EIT-derived physiological patterns.
• The results show that EIT-derived features, supplemented by basic physiological data, can approximate spirometric indicators, highlighting EIT’s potential for continuous monitoring in clinical settings where forced maneuvers are impossible, e.g., intensive care unit, sedated, or ventilated patients.

This is an exploratory predictive study based on a retrospective cohort of 15 adult patients who successfully performed spirometry. Since the patients were examined 2 times, a total of 30 signals were collected. The remainder of this paper is structured as follows: Materials and Methods describes the methodology of the experiment, including a comprehensive analysis of the provided data using statistical and ML methods. Results presents the results, and Discussion discusses them. Finally, Conclusion concludes the paper.

## Materials and Methods

This section provides details regarding data processing and the applied method for data analysis. All experiments were conducted using Python libraries: NumPy (2.4.6), pandas (3.0.3), SymPy (1.14.0), tsfresh (0.21.1), Sweetviz (2.3.3), scikit-learn (1.8.0), Matplotlib (3.10.9), and SHAP (0.51.0).

### Acquisition protocol

The research was conducted in accordance with the opinion of the Ethics Committee of Palacký University Hospital and the Faculty of Medicine and Dentistry, under reference number 13/25. All methods were performed in accordance with the relevant guidelines and regulations. Informed consent was obtained from all patients.

The patients’ chest circumference was measured during both inspiration and expiration. Based on these measurements, the appropriate belt size was selected. The belt was then attached to the patient’s chest, and conductive gel was applied if necessary; this gel was sometimes unnecessary if the patient was not sweating significantly. Following this setup, 3 attempts were made to perform spirometry using the flow-volume method, in accordance with the current European Respiratory Society recommendations and their adaptations in the Czech Republic [20,21]. Spirometry was conducted with the Spirolab MIR device, which had been calibrated for external conditions such as temperature, humidity, and atmospheric pressure. At the same time, EIT was measured and recorded.

### Data processing

**Fig. 1.**
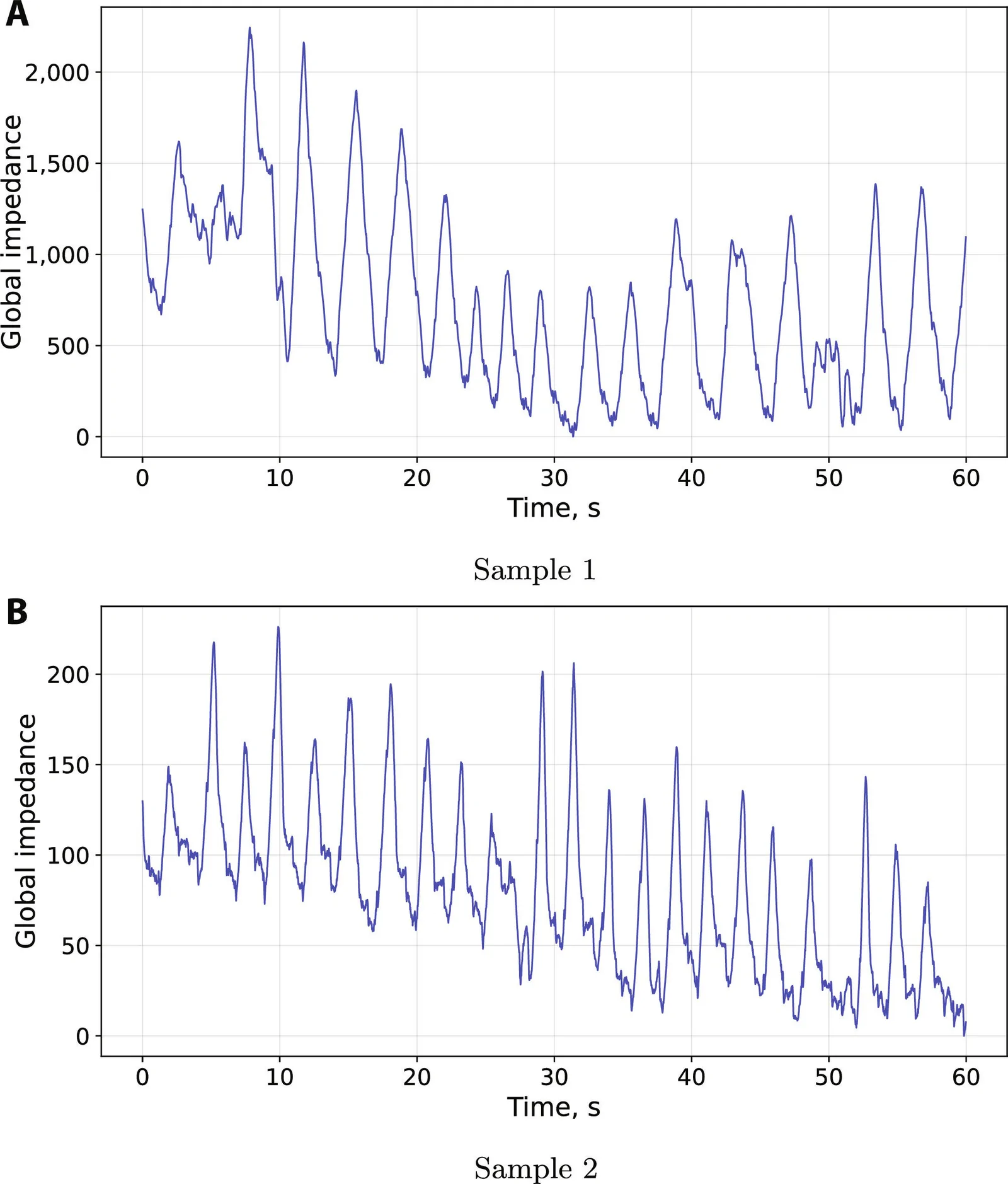
Examples from the dataset. (A) Sample 1. (B) Sample 2.

In this experiment, 30 records from 15 patients were collected using the Dräger EIT software and were provided in .asc file format. Examples of global EIT curves are depicted in Fig. 1. It can be seen that patients are likely breathing spontaneously with varying effort. In the rest of the dataset, some patients showed forced breathing efforts or produced measurement artifacts, such as deep breaths (high peaks), as well as other oscillatory signals that overlapped with physiological noise, for example, heart rate.

For further analysis, numerous time-series characteristics were extracted from global EIT tidal variations using the Python library tsfresh. We focused on the global EIT signal in this exploratory analysis to limit the dimensionality of the feature space and to match the global nature of the Tiffeneau index. Future work will extend the approach to regional indices to better capture ventilation heterogeneity in COPD.

A summary of extracted features is provided in Table 1. Additionally, the dataset includes spirometry values and physiological data of the patients, enabling comprehensive analysis and the execution of various experiments using ML algorithms. In total, the dataset comprises 755 features.

**Table 1.** Summary of feature groups extracted from EIT time series

Feature group	Description
Statistical features	Mean, median, variance, standard deviation, minimum, maximum, range, kurtosis, skewness, coefficient of variation, quantile-based measures
Autocorrelation and partial autocorrelation	Autocorrelation at various lags, aggregated autocorrelation statistics (mean, median, variance across lags), partial autocorrelation coefficients capturing temporal dependency and periodicity
Autoregressive (AR) coefficients	Coefficients of linear autoregressive models of different orders summarizing signal predictability and temporal structure
Frequency-domain features (FFT)	FFT coefficient magnitudes, real/imaginary parts, and phase (angle) components; Fourier energy; Fourier entropy capturing spectral distribution and harmonic structure
Wavelet-based features (CWT)	CWT coefficients across multiple scales, capturing localized time–frequency characteristics and transient respiratory events
Entropy and complexity measures	Approximate entropy, sample entropy, Fourier entropy, and other measures describing the irregularity, complexity, and nonlinearity of the EIT signal
Peak- and shape-based features	Number of peaks, peak amplitude statistics, linear trend coefficients (slope, intercept, *r* value), mass quantile indices, describing breathing waveform morphology
Energy distribution features	Energy ratios computed over fixed temporal segments, capturing uneven ventilation patterns or asymmetric inhalation–exhalation cycles
Outlier and threshold features	Ratio of values beyond rσ, range counts within fixed intervals, identifying extreme fluctuations or unusual signal deviations
Physiological variables	Anthropometric measurements such as body weight and BMI included as baseline clinical predictors

EIT, electrical impedance tomography; FFT, fast Fourier transform; CWT, continuous wavelet transform; BMI, body mass index

The physiological values of patients are introduced by weight, height, BMI, age, and sex. An overview of the given parameters is presented in Table 2, which shows substantial inter-individual variability, particularly in weight and BMI, reflecting the heterogeneity of the study population. The median patient was 88 kg and 176 cm tall and had a BMI of approximately 29.9, placing the central tendency in the overweight range. Ages ranged from 24 to 78 years, with a median of 55, indicating a balanced representation of both younger and older adults.

**Table 2.** Statistics of the physiological values of patients

Statistics	Weight, kg	Height, cm	BMI	Age
Mean	93.67	175.20	30.47	52.08
SD	24.22	6.73	7.48	18.54
Min	56	162	18.00	24
25%	77	174	24.85	34
50%	88	176	29.90	55
75%	111	178	36.10	66
Max	143	186	45.10	78

BMI, body mass index

Although 755 time-series features were extracted, the models were not trained on the full feature set. The small sample size relative to the initial feature space is a key limitation, which we address through feature selection and conservative interpretation.

The examined patients had different diseases, including obstructive sleep apnea syndrome (OSAS) (5), COPD (3), asthma (2), bronchiolitis obliterans (1), and combinations of COPD and OSAS (1) and OSAS, fibrosis, and bronchiolitis obliterans (1), and 2 of them were healthy.

### Statistical analysis methods

This section presents an overview of the statistical techniques used in the experiments and analysis, including *K*-best, *P* value, categorical correlation, and Pearson correlation. These techniques were chosen to perform feature filtering, as a large number of statistical descriptors were derived from the EIT signal, and this selection step helps improve the effectiveness and accuracy of subsequent ML processing. In addition, these methods indicate the relevance of the extracted values with respect to the target Tiffeneau index.

#### *K*-best

The *K*-best feature selection method [22] is a popular univariate statistical filtering technique in ML pipelines, used to identify the most informative features prior to model training. Each feature is assessed independently using scoring functions such as the analysis of variance *F*-score, mutual information, or chi-square statistics. The method retains the top *K* features with the highest relevance scores for further analysis [23]. Studies indicate that univariate filter methods like *K*-best are computationally efficient and robust for high-dimensional data, making them effective as a preprocessing step before applying more complex models [24].

This method is frequently used alongside model-based explainability techniques like Shapley additive explanations (SHAP), as well as dimensionality reduction methods such as principal component analysis, and L1-regularized models to offer deeper insights. Additionally, one approach to data analysis involves creating a pipeline that integrates *K*-best with ML tasks related to lung conditions.

#### *P* value

*P* values are commonly used in medical research and clinical trials for hypothesis testing. They measure the probability that an observed result is due to random chance, assuming that the null hypothesis is true. A smaller *P* value indicates greater statistical significance, with values below 0.05 often considered “statistically significant”. However, this threshold is frequently misinterpreted.

It is important not to rely solely on a fixed *P*-value threshold, such as 0.05, for scientific decision-making and conclusions. Instead, it is advised to concentrate on evaluating the significance of the results by considering the study’s design, the quality of the measurements, and data validity [25].

#### Pearson correlation

The Pearson correlation coefficient (*r*) measures the strength and direction of the linear relationship between 2 quantitative variables, ranging from −1 to +1. A positive correlation (r>0) means that both variables increase together, while a negative correlation (r<0) indicates that as one variable increases, the other decreases. A coefficient of 0 shows no linear relationship, and values close to −1 or +1 denote a strong relationship, whereas values near 0 indicate a weak relationship. This relationship can be expressed by the equation [26]

$$r=\frac{\sum \left(x_{i}-\hat{x}\right)\left(y_{i}-\hat{y}\right)}{\sqrt{\sum {\left(x_{i}-\hat{x}\right)}^{2}}\sqrt{\sum {\left(y_{i}-\hat{y}\right)}^{2}}}.$$ (1)

#### Categorical correlation

The correlation ratio is a statistical measure that assesses the relationship between a categorical variable and a numerical variable, ranging from 0 to 1. It compares the variance between the groups defined by the categorical variable to the total variance of the numerical variable. A value close to 1 indicates distinct groups, while a value near 0 suggests little to no relationship. This serves as an alternative to Pearson’s correlation, which is limited to numerical variables.

## Results

In this work, we focused on 2 complementary scenarios on this dataset: a statistical analysis of all features (case 1) and a predictive analysis using a selected subset of features without spirometry data (case 2).

### Case 1: Statistical analysis for all features

The first part of the analysis utilized pure statistical methods for processed EIT signals and corresponding physiological values. Firstly, the Pearson correlations were computed for each feature with respect to the Tiffeneau index. Table 3 shows the highest correlated features. BMI (0.65) and weight (0.63) showed moderate to strong positive correlations. Several autocorrelation, FFT angle coefficients, and energy-ratio features were negatively correlated (ranging from −0.39 to −0.54), including partial autocorrelation lag 5 (−0.54) and energy ratio by chunks (−0.50).

**Table 3.** Pearson correlations with respect to the Tiffeneau index

Feature	Corr
BMI	0.65
Weight	0.63
Partial autocorrelation lag 5	−0.54
FFT coefficient attr “angle” coeff 2	−0.54
Partial autocorrelation lag 4	−0.50
Energy ratio by chunks num segments 10 segment focus 6	−0.50
FFT coefficient attr “angle” coeff 12	−0.46
FFT coefficient attr “angle” coeff 32	−0.46
Number peaks n 50	−0.46
Ratio beyond r sigma r 3	−0.45
FFT coefficient attr “angle” coeff 80	−0.43
Energy ratio by chunks num segments 10 segment focus 3	−0.41
FFT coefficient attr “angle” coeff 11	−0.39
Agg autocorrelation f agg “median” maxlag 40	−0.39

BMI, body mass index; FFT, fast Fourier transform

A similar analysis was performed using categorical correlation, and the results are presented in Table 4. The highest-ranked features included count below t 0 (0.72), Fourier entropy bins 3 and 10 (0.69 and 0.62, respectively), and length (0.57).

**Table 4.** Categorical ratios with respect to the Tiffeneau index

Feature	Correlation
Count below t 0	0.72
Fourier entropy bins 3	0.69
Fourier entropy bins 10	0.62
Length	0.57
Fourier entropy bins 5	0.50
Sum of reoccurring values	0.45
Sum of reoccurring data points	0.45
Minimum	0.42
Range count max 0	0.42
Count above t 0	0.42
Range count max 1	0.38
Number crossing m 0	0.37
Number crossing m 1	0.29
Number crossing m 2	0.29

The last statistical test evaluated the uncorrected *P* value for feature association, with results presented in Table 5. The top-ranking features (nominal P<0.01) included BMI, weight, and partial autocorrelation lags, while moderately associated features (nominal 0.01<P≤0.05) reflected a range of FFT angle coefficients and entropy bins.

**Table 5.** *P* values for the most statistically significant features with respect to the Tiffeneau index

Column	*P* value
BMI	0.000107
Weight	0.000203
Partial autocorrelation lag 5	0.001942
FFT coefficient attr “angle” coeff 2	0.002053
Partial autocorrelation lag 4	0.002277
Energy ratio by chunks num segments 10 segment focus 6	0.004471
Fourier entropy bins 3	0.005374
FFT coefficient attr “angle” coeff 12	0.010133
FFT coefficient attr “angle” coeff 32	0.010313
Number peaks n 50	0.010502
Ratio beyond r sigma r 3	0.012609
FFT coefficient attr “angle” coeff 80	0.017399
Fourier entropy bins 10	0.018480
Minimum	0.022948
Energy ratio by chunks num segments 10 segment focus 3	0.024840
Fourier entropy bins 5	0.025335
FFT coefficient attr “angle” coeff 11	0.031738
Agg autocorrelation f agg “median” maxlag 40	0.032581
Index mass quantile q 0.1	0.034001
Agg autocorrelation f agg “mean” maxlag 40	0.037697
FFT coefficient attr “angle” coeff 50	0.037966
Ar coefficient coeff 8 k 10	0.039135
FFT coefficient attr “angle” coeff 1	0.042861
Energy ratio by chunks num segments 10 segment focus 5	0.047442
Partial autocorrelation lag 9	0.049525

BMI, body mass index; FFT, fast Fourier transform

**Fig. 2.**
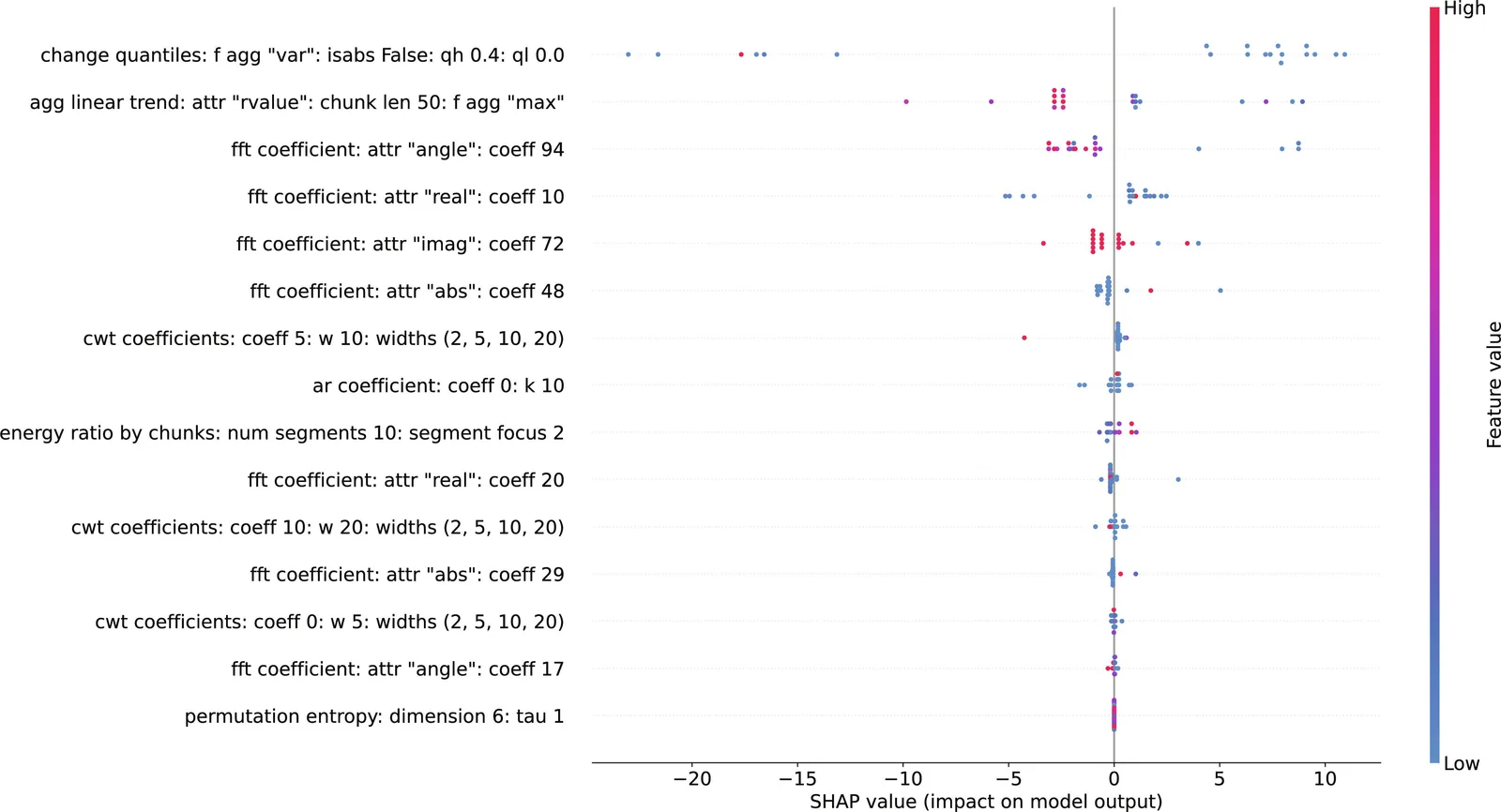
Shapley additive explanations (SHAP) values obtained by experiment, where all features are involved (case 1).

It is critical to note that the *P* values reported in Table 5 were computed without applying a multiple-testing correction. In the context of this study, these *P* values are strictly exploratory and hypothesis generating. They are utilized here solely as a heuristic filtering metric to identify and rank potentially relevant physiological signals prior to the ML and SHAP analyses. To assess feature impact across the entire unselected dataset, SHAP values were computed using a decision tree (DT) trained on all 755 features (Fig. 2). The highest-impact features were derived from FFT attributes, wavelet coefficients, autoregressive models, and energy-ratio measures.

### Case 2: Predictive modeling on selected features

The second case involved feature selection prior to modeling. The features were chosen using the *K*-best feature selection method based on the returned *F*-statistic. The selection was conducted on the full dataset. To ensure reproducibility and explicitly define the modeling configurations, Table 6 details the hyperparameter search spaces utilized for the random search cross-validation. This broad search space allowed the optimizer to navigate the high-dimensional, limited-sample data.

**Table 6.** Hyperparameter search space for ML models evaluated via random search cross-validation

Model	Hyperparameter	Search space
MLP	solver	[‘lbfgs’, ‘sgd’, ‘adam’]
	hidden_layer_sizes	Integers from 2 to 199
	max_iter	Integers from 2 to 149
	activation	[‘identity’, ‘logistic’, ‘tanh’, ‘relu’]
DT	max_features	Integers from 1 to 19
	max_depth	Integers from 1 to 9
	min_samples_leaf	Integers from 1 to 19
RF	n_estimators	$\left\{1,4,7,10,\;\ldots ,\;58\right\}$ (step: 3)
	max_features	Integers from 1 to 14
	max_depth	Integers from 2 to 9
	criterion	[‘squared_error’, ‘absolute_error’, ‘poisson’]
AdaBoost	learning_rate	10 evenly spaced values from 0.0 to 1.0
	n_estimators	Integers from 1 to 29
	loss	[‘linear’, ‘square’, ‘exponential’]
kNN	n_neighbors	Integers from 2 to 29
	weights	[‘uniform’, ‘distance’]
	algorithm	[‘auto’, ‘ball_tree’, ‘kd_tree’, ‘brute’]

ML, machine learning; MLP, multilayer perceptron; DT, decision tree; RF, random forest; kNN, *k*-nearest neighbors

Only 40 features were retained for further analysis to mitigate the high dimensionality of the dataset. The selected features are presented in Table 7.

**Table 7.** Features selected with statistical methods

Feature	Feature
Variation coefficient	Minimum
Autocorrelation lag 9	Agg autocorrelation f agg “mean” maxlag 40
Agg autocorrelation f agg “median” maxlag 40	Agg autocorrelation f agg “var” maxlag 40
Partial autocorrelation lag 4	Partial autocorrelation lag 5
Partial autocorrelation lag 9	Number peaks n 50
Index mass quantile q 0.1	Ar coefficient coeff 1 k 10
Ar coefficient coeff 8 k 10	FFT coefficient attr “angle” coeff 1
FFT coefficient attr “angle” coeff 2	FFT coefficient attr “angle” coeff 6
FFT coefficient attr “angle” coeff 11	FFT coefficient attr “angle” coeff 12
FFT coefficient attr “angle” coeff 32	FFT coefficient attr “angle” coeff 37
FFT coefficient attr “angle” coeff 50	FFT coefficient attr “angle” coeff 53
FFT coefficient attr “angle” coeff 59	FFT coefficient attr “angle” coeff 76
FFT coefficient attr “angle” coeff 80	FFT coefficient attr “angle” coeff 88
Range count max 1 min −1	Approximate entropy m 2 r 0.9
Energy ratio by chunks num segments 10 segment focus 0	Energy ratio by chunks num segments 10 segment focus 3
Energy ratio by chunks num segments 10 segment focus 4	Energy ratio by chunks num segments 10 segment focus 5
Energy ratio by chunks num segments 10 segment focus 6	Ratio beyond r sigma r 2.5
Ratio beyond r sigma r 3	Fourier entropy bins 3
Fourier entropy bins 5	Fourier entropy bins 10
Weight	BMI

FFT, fast Fourier transform; BMI, body mass index

Based on these selected features, several ML algorithms were applied. The dataset was split into training and testing sets in a 2:1 ratio. The patient-level split was also considered to preserve the measurements from one patient in only the train or test set. Random search with cross-validation (*k* = 3) and 100 iterations was performed on the training set to identify optimal hyperparameters. The final performance on the testing set is presented in Table 8, utilizing mean squared error (MSE) and the $R^{2}$ score.

**Table 8.** Model performance comparison sorted by $R^{2}$

Model	MSE	$R^{2}$	Best parameters
MLP	56.16	0.72	solver: sgd; max iterations: 41; hidden layer sizes: 144; activation: logistic
DT	67.39	0.66	min samples leaf: 1; max features: 2; max depth: 2
AdaBoost	79.08	0.60	number of estimators: 13; loss: ‘linear’; learning rate: 1.0
RF	143.38	0.28	number of estimators: 7; max features: 4; max depth: 8; criterion: poisson
kNN	147.51	0.26	weights: uniform; number of neighbors: 9; algorithm: brute
Linear regression	337.33	−0.70	–

MSE, mean squared error; MLP, multilayer perceptron; DT, decision tree; RF, random forest; kNN, *k*-nearest neighbors

**Fig. 3.**
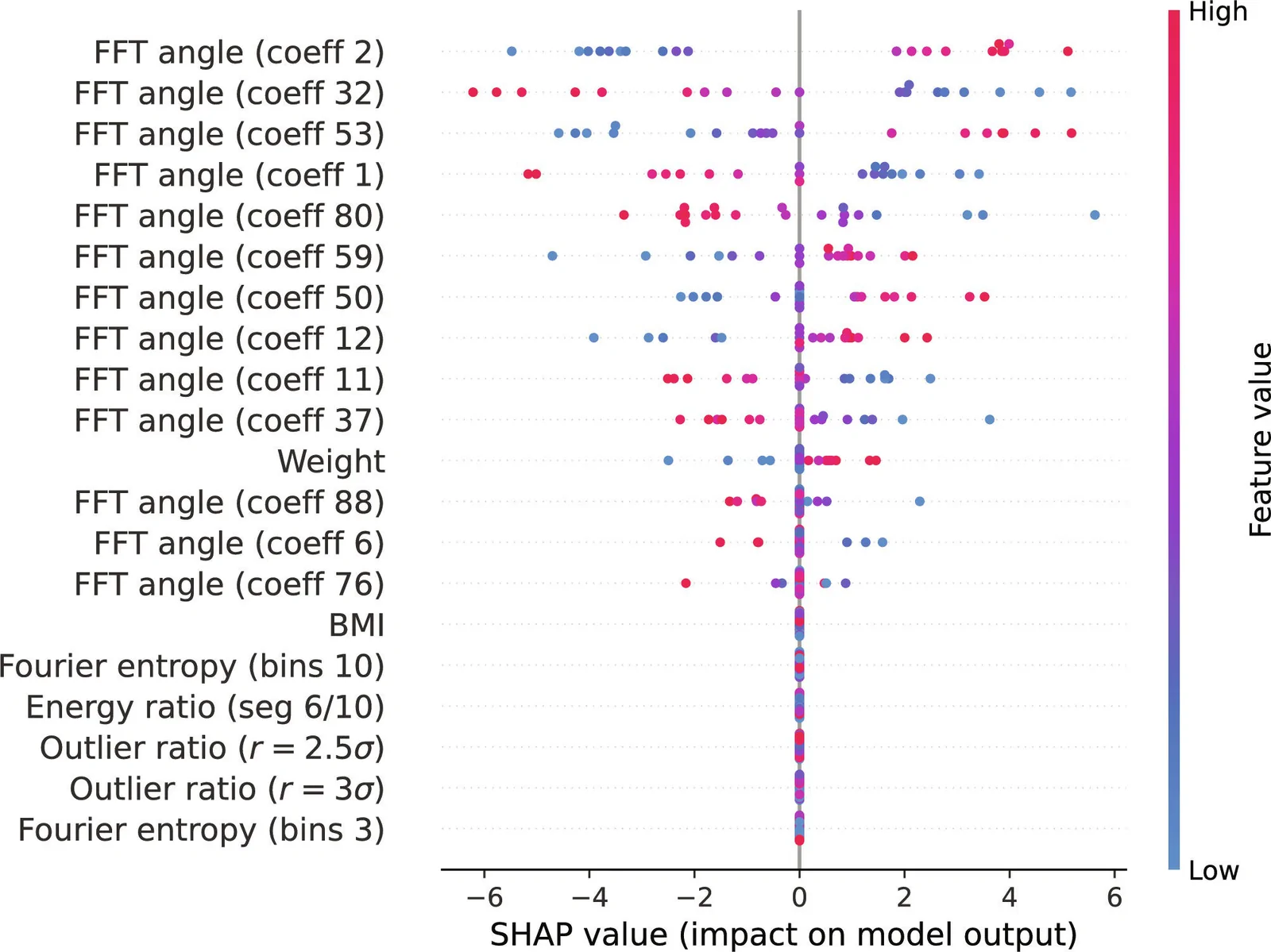
Shapley additive explanations (SHAP) values for selected features.

**Fig. 4.**
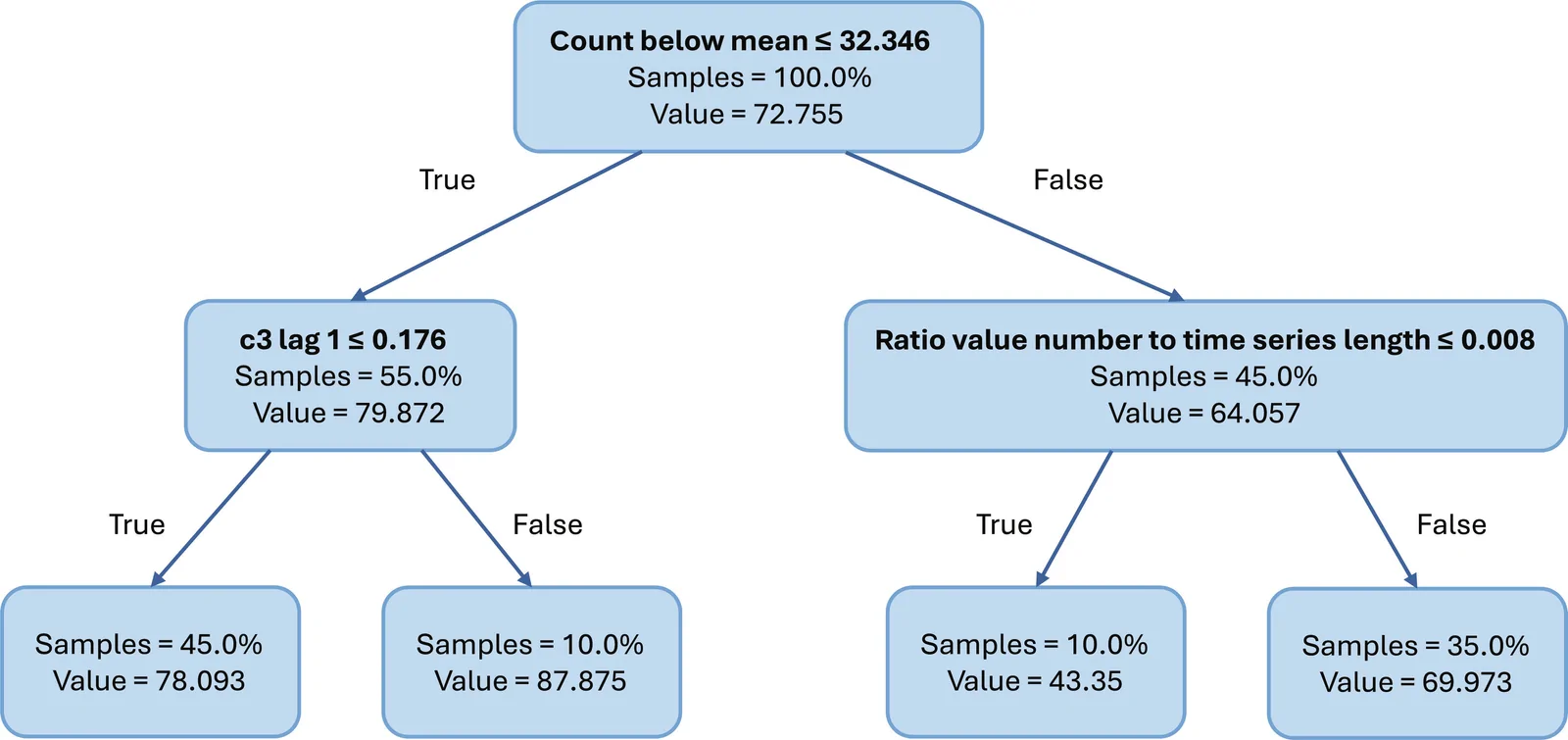
Decision tree for selected features.

The MLP achieved the highest numeric performance ($R^{2}=0.72$, MSE = 56.16), followed by the DT and AdaBoost. Figure 3 visualizes the feature impact for the MLP output using the SHAP method, highlighting the prominent role of FFT angles and anthropometric factors. Figure 4 illustrates the structure of the resulting DT model for interpretability.

### Ablation study

Given the strong univariate correlations observed between the Tiffeneau index and anthropometric variables such as BMI and weight, a critical concern is whether the ML models merely predict body habitus rather than true underlying lung function. To address this potential confounding effect and explicitly evaluate the incremental predictive value of the EIT-derived physiological signals, an ablation study was performed. Using the best-performing MLP architecture, we compared the predictive performance of 3 distinct feature sets: (a) clinical-only, comprising standard anthropometric baseline data (e.g., age, sex, weight, height, and BMI); (b) EIT-only, restricting the input strictly to the 38 EIT-derived time-series features identified by the *K*-best selection; and (c) clinical + EIT, combining the selected EIT features with the important clinical variables. The results are summarized in Table 9.

**Table 9.** Ablation study for case 2. Bold text indicates the best results.

Feature set	Num. of feat.	Test $R^{2}$	Test RMSE	Test MAE
Clinical-only	5	−0.119	14.921	10.499
EIT-only	38	0.713	7.555	6.007
**Clinical + EIT**	**40**	**0.718**	**7.494**	**5.211**

RMSE, root mean square error; MAE, mean absolute error; EIT, electrical impedance tomography

The results clearly demonstrate that baseline clinical and anthropometric variables alone are entirely insufficient for predicting the Tiffeneau index. The clinical-only model failed to generalize, yielding a negative $R^{2}$ (−0.119) and a severely inflated root mean square error (RMSE) of 14.921, indicating that the model performed worse than a simple mean predictor. This confirms that while body mass correlates with the target variable, it lacks the functional physiological information required to accurately estimate airflow limitation on its own. However, removing clinical data entirely and relying strictly on the 38 EIT-only features yielded an $R^{2}$ of 0.713 and an RMSE of 7.555. This performance is nearly identical to that of the fully combined model, suggesting that most of the predictive signal is carried by the EIT temporal and spectral features (e.g., FFT phase coefficients, entropy, and autocorrelation). Finally, the combined Clinical + EIT model achieved the highest overall performance ($R^{2}=0.718$, RMSE = 7.494), indicating that while EIT data drive the core prediction of respiratory mechanics, anthropometric variables offer a slight complementary benefit, likely by helping the model scale or calibrate the baseline impedance signals to individual patient body sizes. This ablation analysis confirms that the proposed framework successfully leverages EIT-specific physiological information rather than merely acting as a proxy for body habitus.

### Model robustness analysis

Given the high-dimensional nature of the feature space relative to the small sample size (the p≫n regime), it is important to assess how model performance generalizes across different data subsets. To rigorously evaluate the stability of our tuned models, we subjected them to repeated cross-validation (Repeated KFold with n_splits = 3 and n_repeats = 20, yielding 60 validation folds per model). This approach provides a comprehensive view of the expected mean performance, alongside the empirical 95% confidence intervals (2.5th and 97.5th percentiles). The results are summarized in Table 10.

**Table 10.** Robustness analysis for all tuned models

Model	Mean $R^{2}$	$R^{2}$ SD	$R^{2}$ 2.5%	$R^{2}$ 97.5%	Mean MAE	Mean RMSE
MLP	0.169	0.683	−1.663	0.726	8.185	10.240
kNN	0.139	0.505	−1.223	0.500	9.064	11.356
AdaBoost	0.094	0.634	−1.475	0.729	9.317	11.325
RF	0.079	0.596	−1.577	0.671	9.424	11.412
DT	−0.182	0.893	−2.717	0.732	9.897	12.632
Linear regression	−2.665	5.030	−17.347	0.531	15.901	19.693

MAE, mean absolute error; RMSE, root mean square error; MLP, multilayer perceptron; kNN, *k*-nearest neighbors; RF, random forest; DT, decision tree

The repeated cross-validation demonstrates that the MLP maintains the highest mean $R^{2}$ (0.169) and the lowest mean RMSE (10.240) across all variations of the dataset. Notably, the upper bound of the MLP’s performance reaches an $R^{2}$ of 0.726, confirming that when the training split adequately captures the underlying physiological distribution, the EIT features hold substantial predictive power. Furthermore, all nonlinear ML models maintained positive mean $R^{2}$ scores, significantly outperforming standard linear regression, which struggled to capture the complex relationships in the data. As expected in an exploratory p≫n setting, the variance across folds is relatively high. Permutation testing with 100 permutations supported this interpretation: while the observed performance on optimal splits captures genuine physiological patterns, the small sample size limits the ability to achieve uniform statistical significance across all random folds. This indicates a high sensitivity to the training data distribution.

## Discussion

This exploratory study investigated the feasibility of estimating the Tiffeneau index (FEV1/FVC) using purely time-series features extracted from global EIT tidal breathing signals, without requiring forced expiratory maneuvers. Our initial results suggest that combining EIT-derived temporal and spectral features with basic anthropometrics yields mathematically strong correlations with this key spirometric indicator. However, given the extremely high-dimensional feature space (p≫n) relative to our small sample size (15 patients, 30 measurements), these findings should be interpreted with careful consideration of both methodological and clinical limitations.

### Model performance

The contrasting performance of the DT ($R^{2}=0.66$) and random forest (RF) ($R^{2}=0.28$) highlights the challenges of modeling our limited-sample, high-dimensional (p≫n) dataset. The success of a highly constrained, shallow DT (max_depth = 2) indicates that the predictive signal is heavily dominated by a few high-impact features. The tree easily isolates these variables at its root splits, capturing the bulk of the variance, while the MLP ($R^{2}=0.72$) merely captures additional nonlinearities to slightly improve accuracy. Conversely, the lower performance of the RF highlights the well-known behavior of ensemble methods in datasets where the predictive power is highly concentrated. Because an RF relies on data bootstrapping and random feature subsetting, individual trees in the ensemble are frequently forced to split on secondary features rather than the dominant predictors. This dynamic is directly reflected in the RF hyperparameters (7 estimators, Poisson criterion) identified through our systematic random search (Table 6). Rather than defaulting to a large, standard ensemble, the optimization process correctly adapted by selecting a highly compact forest.

### Predictive potential and methodological considerations

The results of the repeated cross-validation (Table 10) provide valuable, transparent insights into the feasibility of applying ML to EIT signals. The ability of the MLP to achieve an $R^{2}$ of up to 0.726 on favorable data splits is a highly promising indicator. It mathematically confirms our primary hypothesis: EIT-derived time-series features, specifically phase and entropy descriptors, do encode clinically relevant information about expiratory airflow limitations without requiring forced breathing maneuvers. However, the mean $R^{2}$ of 0.169 and the accompanying variance also highlight the inherent challenges of modeling in a p≫n regime. With 40 features and only 30 measurements, the data manifold is too sparse to consistently anchor the weights of a high-capacity model across every possible data split. This variance does not invalidate the predictive signal. Rather, it underscores that a larger, more comprehensive dataset is required to stabilize the models’ decision boundaries and prevent overfitting. From a clinical precision standpoint, the mean RMSE of approximately 10.24 on the Tiffeneau index is an encouraging baseline for a completely noninvasive, effort-free measurement, especially considering the natural variability of standard spirometry. Nevertheless, because the diagnostic threshold for airflow obstruction is strictly defined at 70%, an error margin of this size could occasionally straddle the boundary between a healthy and an obstructed classification. Therefore, in its current state of development, this EIT-based framework is best viewed not as a standalone replacement for point-in-time diagnostic spirometry but as a powerful foundational tool. It shows big promise for continuous, trend-based monitoring, where tracking relative changes in respiratory mechanics is more critical than absolute threshold precision, and paves the way for larger validation studies to fully unlock the predictive power of EIT features.

### Clinical precision and practical application

While an $R^{2}$ of 0.72 indicates a statistical relationship, the clinical utility of the model must be evaluated through its absolute error margins. The best model’s MSE of 56.16 corresponds to an RMSE of approximately 7.5 percentage points on the Tiffeneau index. Because the diagnostic threshold for airflow obstruction is strictly defined at 70%, an error margin of ±7.5% straddles this clinically decisive boundary. For instance, a patient with a true index of 65% (obstructed) could easily be predicted at 72% (normal), leading to a missed diagnosis. Therefore, at its current precision, this EIT-based approach is better suited as a continuous monitoring tool to track relative changes in respiratory mechanics over time, rather than a definitive replacement for point-in-time diagnostic spirometry.

### Feature interpretation and biological relevance

A core goal of this study was to move beyond black-box predictions by interpreting the features that drive the model. Our statistical and SHAP analyses consistently highlighted FFT angle (phase) coefficients, entropy, and autocorrelation as top predictors. While the dominance of FFT phase coefficients might initially seem physiologically counterintuitive compared to amplitude or power, phase holds critical temporal information. Obstructive lung diseases alter the timing and symmetry of the respiratory cycle, classically causing prolonged expiration. FFT phase coefficients capture this time–frequency alignment and asymmetry in the respiratory waveform. Similarly, altered entropy and autocorrelation point to less complex, more uniformly repetitive signal structures, which may correlate with the restricted mechanical dynamics of impaired lungs. Nevertheless, we must acknowledge that the FFT phase is notoriously sensitive to signal windowing and alignment. Given the lack of a standardized physical context in the automated feature search, we cannot definitively rule out that the predictive power attributed to these specific phase coefficients represents a fit to physiological noise.

### The confounding role of anthropometrics and overfitting caveats

It is vital to address the strong predictive influence of BMI and weight. The confounding influence of anthropometric variables on physiological or anatomical measurements has been documented in work by Zanfardino *et al.* [27]. In a large cardiac computed tomography cohort study, unsupervised RF clustering identified BMI and body surface area as the dominant drivers of patient subgrouping, to the extent that reference ranges for ascending aorta diameter differed substantially between BMI-defined clusters. This finding underscores the importance of explicitly accounting for body-size covariates before attributing a predictive signal to a target physiological measurement. Since BMI and weight are among the strongest correlates of the Tiffeneau index in our dataset, there is a risk that the models may be partially predicting body habitus rather than solely lung function. Furthermore, the SHAP values computed in case 1 (Fig. 2) were derived from a DT trained on the full set of 755 features using approximately 30 samples. Such a model is severely overfitted. The feature-importance rankings for case 1 should not be viewed as final or definitive. They come with important warnings and should only be seen as a starting point for generating hypotheses.

### Comparison with prior literature

Previous studies have established foundational evidence for using EIT to estimate lung function, exploring both statistical correlations and predictive modeling. For instance, investigations into the spectral properties of EIT signals in COPD patients have identified specific frequency bands with strong associations to FVC, FEV1, and the Tiffeneau index. Such analyses have achieved correlations exceeding 60%, suggesting that integrating anthropometric data with advanced signal modeling could further enhance predictive accuracy [4]. The feasibility of noninvasive respiratory assessment has been further supported by portable, self-administrable EIT systems, which have demonstrated strong correlations for spirometric indices in healthy subjects and provided spatial insights into ventilation distribution for potential home monitoring [12]. When extending these concepts to more robust predictive modeling, Zouari *et al.* [9] utilized simultaneous EIT and spirometry data from healthy subjects performing varied breathing efforts. Their regression models, which combined EIT-derived features with biometrics, achieved correlations above 0.68 for all key indicators, alongside up to 98% sensitivity and 100% specificity for detecting obstructive patterns. Moving beyond linear regression, recent works have employed ML frameworks to automate this estimation. By extracting frequency, Fourier entropy, and autocorrelation metrics from EIT signals and combining them with clinical parameters such as BMI and weight, these models have achieved classification accuracies of approximately 0.60 for the Tiffeneau index without requiring direct, simultaneous spirometry input [5,10]. In our testing, the MLP achieved $R^{2}=0.72$, which nominally aligns with or exceeds the predictive potential demonstrated in these prior studies. However, the credibility of this specific point estimate must be tempered. Training a high-capacity nonlinear model (144 hidden neurons) on an exceedingly small dataset carries a severe risk of overfitting. While the MLP achieved the lowest MSE on this specific test set, this does not serve as definitive evidence that complex neural networks are universally better suited for predicting lung function from EIT than simpler linear or tree-based baselines. The variations in performance across models, such as the poor generalization of the RF compared to the shallow DT, likely reflect instability due to the microscopic sample size rather than underlying physiological truths.

### Limitations

The present study has several important limitations to consider. First, the small sample size increases the risk of overfitting, resulting in uncertainty around performance metrics. Second, the clinical characterization of the cohort is limited, with incomplete data on COPD stage, smoking status, and comorbidities, affecting generalizability and the separation of disease severity from other factors. Third, the analysis uses the global EIT signal instead of regional indices, which may overlook important spatial ventilation patterns in the heterogeneous nature of COPD and other obstructive diseases. It should also be mentioned that our cohort is small (*n* = 15) and highly heterogeneous (including OSAS, asthma, and COPD). Because only a small subset of patients exhibited healthy, severe obstruction, the framing of these results as a definitive COPD diagnostic tool is limited. The findings currently reflect generalized respiratory variability rather than definitive disease characterization. Fourth, strong predictors such as BMI and weight may indicate that some predictive signals are influenced by body size rather than lung physiology, with the small dataset limiting adjustments for these factors. Finally, performance estimates are based on internal resampling without external validation, likely leading to overestimation of real-world performance and limiting the findings’ applicability for clinical deployment. The results should be viewed as a preliminary step requiring further validation in larger and more diverse populations.

## Conclusion

This exploratory study investigated whether EIT-derived signal features, in conjunction with basic physiological data, may be associated with the Tiffeneau index (FEV1/FVC), a key spirometric parameter for pulmonary function. The analysis was conducted in 2 cases: using all features extracted from EIT data and using a selected subset of features. In this small cohort, both clinical variables (weight and BMI) and signal-derived descriptors (FFT phase coefficients, entropy, and autocorrelation) appeared among the strongest contributors to model predictions, and the alignment between SHAP-based feature importance and prior univariate association analysis suggests some consistency in the identified signal patterns. However, given the high feature-to-sample ratio (up to 755 candidate features from only 15 patients and 30 measurements), these associations should be regarded as preliminary and hypothesis generating rather than confirmed predictive relationships. Notably, BMI and weight were among the strongest correlates of the Tiffeneau index, raising the possibility that part of the observed predictive signal reflects body-size confounding rather than EIT-specific physiological information. This distinction could not be fully disentangled with the present sample size. Taken together, these exploratory findings suggest that EIT-derived temporal and spectral features, combined with basic anthropometrics, may hold potential for noninvasive estimation of the Tiffeneau index. However, they do not yet constitute confirmed evidence of predictive validity. Given the limited sample size, the absence of patient-level external validation, potential anthropometric confounding, and the lack of data from patients unable to perform spirometry, these results should be interpreted strictly as a feasibility signal rather than evidence supporting clinical deployment. Future work should prioritize larger, prospectively validated cohorts, ideally including the target population unable to undergo conventional spirometry, using patient-level grouped validation and ablation analyses to isolate the incremental contribution of EIT features beyond anthropometric covariates.

## Ethical Approval

The research was conducted in accordance with the opinion of the Ethics Committee of Palacký University Hospital and the Faculty of Medicine and Dentistry, under reference number 13/25.

## Data Availability

Data are available on request.
